# Medicines use before and after comprehensive medicines review among residents of long-term care facilities: a retrospective cohort study

**DOI:** 10.1186/s12877-022-03187-0

**Published:** 2022-06-08

**Authors:** Janet K. Sluggett, Gillian E. Caughey, Tracy Air, Max Moldovan, Catherine Lang, Grant Martin, Stephen R. Carter, Shane Jackson, Andrew C. Stafford, Steve L. Wesselingh, Maria C. Inacio

**Affiliations:** 1 University of South Australia, UniSA Allied Health and Human Performance, GPO Box 2471, Adelaide, South Australia Australia; 2 Registry of Senior Australians, South Australian Health and Medical Research Institute, Adelaide, South Australia Australia; 3 Centre for Medicine Use and Safety, Faculty of Pharmacy and Pharmaceutical Sciences, Monash University, Parkville, VIC Australia; 4 Biometry Hub, Faculty of Sciences, Engineering and Technology, The University of Adelaide, Waite Campus, Urrbrae, South Australia Australia; 5 Australian Association of Consultant Pharmacy, Australian Capital Territory, Fyshwick, Australia; 6 School of Pharmacy, Faculty of Medicine and Health, The University of Sydney, Sydney, NSW Australia; 7 School of Pharmacy and Pharmacology, University of Tasmania, Hobart, TAS Australia; 8 Curtin Medical School, Faculty of Health Sciences, Curtin University, Perth, WA Australia

**Keywords:** Medication review, Medication therapy management, Pharmacists, Drug utilization, Long-term care, Nursing homes, Residential facilities, Homes for the aged, Australia, Residential aged care

## Abstract

**Background:**

Residential Medication Management Review (RMMR) is a subsidized comprehensive medicines review program for individuals in Australian residential aged care facilities (RACFs). This study examined weekly trends in medicines use in the four months before and after an RMMR and among a comparison group of residents who did not receive an RMMR.

**Methods:**

This retrospective cohort study included individuals aged 65 to 105 years who first entered permanent care between 1/1/2012 and 31/12/2016 in South Australia, Victoria, or New South Wales, and were taking at least one medicine. Individuals with an RMMR within 12 months of RACF entry were classified into one of three groups: (i) RMMR within 0 to 3 months, (ii) 3 to 6 months, or (iii) within 6 to 12 months of RACF entry. Individuals without RMMRs were included in the comparison group. Weekly trends in the number of defined daily doses per 1000 days were determined in the four months before and after the RMMR (or assigned index date in the comparison group) for 14 medicine classes.

**Results:**

113909 individuals from 1979 RACFs were included, of whom 55021 received an RMMR. Across all three periods examined, decreased use of statins and proton pump inhibitors was observed post-RMMR in comparison to those without RMMRs. Decreases in calcium channel blockers, benzodiazepines/zopiclone, and antidepressants were observed following RMMR provision in the 3–6 and 6–12 months after RACF entry. Negligible changes in antipsychotic use were also observed following an RMMR in the 6–12 months after RACF entry by comparison to those without RMMRs. No changes in use of opioids, ACE inhibitors/sartans, beta blockers, loop diuretics, oral anticoagulants, or medicines for osteoporosis, diabetes or the cognitive symptoms of dementia were observed post-RMMR.

**Conclusions:**

For six of the 14 medicine classes investigated, modest changes in weekly trends in use were observed after the provision of an RMMR in the 6–12 months after RACF entry compared to those without RMMRs. Findings suggest that activities such as medicines reconciliation may be prioritized when an RMMR is provided on RACF entry, with deprescribing more likely after an RMMR the longer a resident has been in the RACF.

**Supplementary Information:**

The online version contains supplementary material available at 10.1186/s12877-022-03187-0.

## Background

Older people living in residential aged care facilities (RACFs) are frequently exposed to high-risk and potentially inappropriate medicines. Between 19 and 22% of residents are exposed to an antipsychotic, 22–52% receive an opioid, 3–58% of residents with diabetes receive insulin and 36–58% of residents with atrial fibrillation receive an anticoagulant, with half of all residents of RACFs exposed to at least one potentially inappropriate medicine [1–5]. While appropriate for some individuals, these medicines are often associated with harms such as drowsiness, confusion, falls and hospitalization, and may necessitate strategies to optimize care such as close monitoring, deprescribing and/or substitution with non-pharmacological therapies [6].

Initiatives to support safe and efficacious medicines use in RACFs include clinical governance structures, standardized medicines administration charts, clinical guidelines, decision support systems, quality indicators, education, case conferencing, and comprehensive medicines reviews [1, 7]. In Australia, the government-subsidized Residential Medication Management Review (RMMR) comprehensive medicines review program has operated since 1997 [8, 9]. In 2019, there were 95491 RMMR claims submitted by pharmacists and the Australian Government spent AUD 18.75 million on pharmacist and general medical practitioner (GP) remuneration for RMMR services [10, 11]. RMMRs involve collaboration between a resident’s usual GP and a pharmacist who has successfully completed a two-stage accreditation process to provide RMMR services. National guidance is available regarding the types of activities that can be provided during an RMMR [9], although accredited pharmacists are encouraged to tailor RMMR activities to the reason for referral and the resident’s care goals. In general, after receiving a GP referral, the pharmacist visits the RACF to review clinical documentation and participate in discussions with the resident, family and/or RACF staff to obtain a best possible medicines history, identify medicines related problems and provide education. RMMR guidelines and medication management guidelines for Australian RACFs both note the importance of medicines reconciliation and review following care transitions such as RACF entry or on return from hospital [9, 12]. An average of three to four medicines related problems (e.g., undertreated conditions, problems with medicine selection such as duplications or drug interactions, adverse drug reactions, need for additional monitoring) are identified per RMMR [13]. The pharmacist provides a report to the GP outlining suggestions to address the medicines-related problems, which the GP reviews and uses to develop and implement a medicines management plan for the resident.

RMMRs are generally recommended when a resident first enters an RACF and when an individual’s clinical circumstances change. The need for broad and consistent implementation of RMMRs was identified during the recent Royal Commission into Aged Care Quality and Safety and as part of Australia’s response to the World Health Organization’s (WHO) Third Global Patient Safety Challenge, *Medication without harm*, which aims to reduce severe, avoidable medicines-related harm by 50% globally over a five-year period [14, 15]. International consensus guidelines also recommend regular medicines reconciliation and review for frail older people to optimize medicines management [16].

Provision of comprehensive medicines reviews in RACFs has been shown to improve appropriateness of medicines use [13, 17]. However, few studies have examined changes in the use of specific medicines following an RMMR. A cluster randomized controlled trial published in 2001 that involved 52 Australian RACFs and tested a multifaceted intervention comprising stakeholder engagement, education and medicines reviews reported reductions in the use of laxatives, medicines for reflux, non-steroidal anti-inflammatory drugs, and benzodiazepines in the intervention arm during follow-up [18]. However, this trial was undertaken prior to the implementation to the current RMMR program and utilized a different model than what is currently in practice. More recently, two small studies reported modest reductions in exposure to anticholinergic and/or sedative medicines following an RMMR [19, 20]. A recent Australian study reported no change in benzodiazepine use in the six months following provision of an RMMR or Home Medicines Review (HMR), a related service for community-dwelling people, but lacked data on access to aged care service use and did not stratify findings by type of medicines review [21]. Despite the 25-year history of the RMMR program in Australia, population-based changes in medicines use following an RMMR have not been examined. Hence, this study examined weekly trends in medicines use in the four months before and after an RMMR in RACFs and among a comparison group of individuals in the RACF who never received an RMMR.

## Methods

### Study design and data sources

A retrospective cohort study was conducted using data from the National Historical cohort of the Registry of Senior Australians (ROSA) [22, 23]. ROSA contains deidentified demographic, clinical, aged care, health service and medicines utilization information for all people aged ≥ 65 years who access government-subsidized residential aged care services. ROSA includes linked aged care data, including data collected during aged care eligibility assessments [24], entry into residential care assessments [25] and aged care service records, together with data from the Australian Institute of Health and Welfare National Death Index. ROSA also includes administrative records providing information about medicines dispensed via the Pharmaceutical Benefits Scheme (PBS) (Australia’s national medicines subsidy scheme) which subsidizes the cost of many approved medicines in Australia, with 906 different medicines (5,380 brand names) listed on the PBS as of 30^th^ June 2021 [26]. ROSA also includes information about GP and other health services accessed through Australia’s Medicare Benefits Schedule (MBS), and hospitalization records from four Australian states’ health authorities. Claims for medicines are coded according to the WHO Anatomical Therapeutic Chemical classification system [27] and PBS item codes [26]. MBS claims are coded according to MBS item codes [28]. The Australian Institute of Health and Welfare and the University of South Australia Human Research Ethics Committees approved a waiver of written informed consent for individuals whose data is included in the ROSA National Historical Cohort and utilized in this study as described in the ‘Ethics approval and consent to participate’ section below.

### Study cohort

**Fig. 1:**
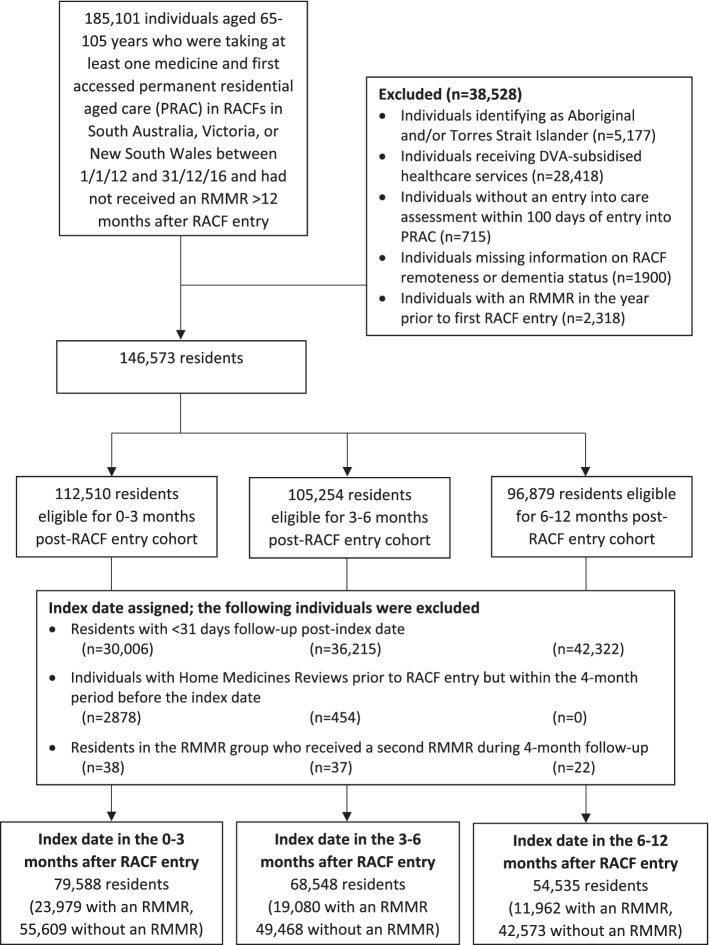
Flow chart describing the study cohort selection. DVA Department of Veterans' Affairs, PRAC permanent residential aged care, RACF residential aged care facility, RMMR residential medication management review

Individuals aged between 65 and 105 years with an aged care eligibility assessment who entered an RACF for the first time between 1st January 2012 and 31 December 2016 in three Australian states (South Australia (SA), Victoria (VIC) or New South Wales (NSW)) and were dispensed at least one government-subsidized (i.e., PBS) medicine in the six months before entry were eligible for inclusion (*n* = 185101, Fig. 1). The date of RACF entry was the date of entry into permanent residential aged care and did not include time spent in temporary respite care because RMMRs were not subsidized for respite care during the period of interest.

The following were excluded: individuals identifying as Aboriginal and/or Torres Strait Islander (due to lack of current ethical approvals), individuals receiving healthcare services subsidized by the Australian Government Department of Veterans’ Affairs, individuals who did not receive an entry into care assessment within 100 days of RACF entry, individuals missing data on RACF remoteness or dementia status (because this was utilized for index date assignment as described below), and those with an RMMR in the year prior to RACF entry (as RMMRs can be provided in certain circumstances such as during transition care) (*n* = 38528, Fig. 1). This left *n* = 146573 individuals eligible for index date assignment, of whom *n* = 113090 met the final inclusion criteria.

### Exposure of interest

The exposure of interest was the first GP MBS claim for a comprehensive medicines review (RMMR MBS item code 903, HMR MBS item code 900) within 12 months of RACF entry. Individuals who received their first RMMR more than 12 months after entry to RACF were not eligible for inclusion. Previous research has shown that the majority of residents who receive an RMMR are provided the service within the first 12 months of RACF entry [28]. Claims for HMRs after RACF entry were also considered to represent RMMRs due to similarities in names and MBS item codes, comprising 3.5% of all comprehensive medicines review claims after RACF entry in previous studies [23, 29].

### Index date assignment for exposed (RMMR) and unexposed (no RMMR)

For residents with an RMMR (exposed group), the index date for the analysis was the date of RMMR provision. These individuals were then classified into three groups: (i) RMMR within 0 to 3 months (1–90 days), (ii) RMMR within 3 to 6 months (91–182 days), or (iii) RMMR within 6 to 12 months (183–365 days) of RACF entry (Supplementary Fig. 1). While RMMRs are recommended at RACF entry, few residents receive an RMMR at this time and considerable changes in medicines use occur around the time of RACF entry [3, 23, 29, 30]. Therefore, examining a wider time-period enabled the identification of individuals receiving an RMMR after the initial transition to an RACF who may be experiencing less changes in medicines and healthcare use.

The index date for individuals without RMMRs was determined by matching to individuals with RMMRs based on five criteria: age at RACF entry (categorized as 65–74, 75–84, 85–94, ≥ 95 years), sex (male, female), diagnosis of dementia (yes, no), remoteness of residence (major city, other), and number of unique prescriptions dispensed in the year prior to RACF entry (categorized as 1–5, 6–10, 11–15, 16–20, ≥ 21 prescriptions dispensed) (i.e., matching into one of 160 unique subgroups). The median time from RACF entry to the first RMMR for each subgroup was assigned as the corresponding index date for unexposed individuals in the same subgroups. The matching described above was only used to assign the index date in the unexposed group and was not used for initial cohort selection or any additional analyses.

Individuals who did not receive an RMMR during their RACF stay (unexposed group) were included in the comparison group provided they were alive at the index date. Unexposed individuals were eligible for inclusion in more than one analysis period, with a different index date for each period if they remained eligible for inclusion.

### Follow-up period

The index date was day 1 of follow-up and individuals were followed for up to 119 days (17 weeks) (i.e., 4 months). Individuals in the RMMR group who received a subsequent RMMR during follow-up were excluded (Fig. 1). All individuals needed to complete a minimum of 4 weeks follow-up. Thereafter, residents that died or left the RACF for another reason were censored (i.e., removed from the analysis) and thus contributed data up until the date of RACF departure.

### Outcomes of interest

The outcome of interest was medicines use in the four months (17 weeks) before and four months after the index date, for 14 medicine classes (Supplementary Table 1). The medicines of interest were selected a priori based on the most common utilized medicine classes and health conditions among residents included in previous Australian RACF studies [22, 31, 32] and the availability of PBS subsidies. Psychotropic medicines were included as per concerns raised by the recent Royal Commission into Aged Care Quality and Safety [15]. The outcome was quantified by the number of defined daily doses (DDDs) of the 14 specific medicine classes per 1000 resident-days per week. The DDD for a medicine reflects the average maintenance dose when the medicine is used for its main indication in an adult [27].

To determine weekly medicines exposure, estimated prescription durations were applied to medicines claims data in the year prior to the index date and for 17 weeks (119 days) after the index date (Supplementary Fig. 2). The waiting time distribution [33] for each medicine was used to estimate the duration of use for each prescription dispensed as dosing information is not recorded in PBS claims data. Individuals were considered as taking the medicine from the date of dispensing plus the length of the prescription duration estimate. Each time a medicine of interest was dispensed, the number of DDDs available for use each day was calculated by dividing the total number of DDDs dispensed by the prescription duration. The 17-week period was selected to reflect changes around the time of RMMR provision and was informed by the usual prescription duration for the medicines examined. Many prescription durations were < 17 weeks, meaning the majority of medicines dispensed immediately prior to the RMMR would be considered depleted prior to the end of the 17-week follow-up period.

### Statistical analysis

Descriptive statistics were used to describe the study cohort stratified by the index date relative to RACF entry (i.e., 0–3 months, 3–6 months, and 6–12 months after RACF entry).

Medicines use in the 17 weeks before and after the index date was plotted graphically for exposed and unexposed individuals for each of the index date groups. Significant variability in medicines use during the transition into an RACF was observed for individuals with an index date in the 0–3 months or 3–6 months after RACF entry; consequently, only individuals with an index date in the 6–12 months after RACF entry were modelled further.

For those with an index date during the 6–12 months after RACF entry, controlled interrupted time series (CITS) analyses were performed using segmented regression modelling. The segmented regression examined three periods: i) the 17-week pre-intervention period (17 time-points before the index date), ii) the washout period (a 5-week period commencing at the index date; 5 time-points), and iii) the follow-up period (from weeks 6 to 17 after the index date; 12 time-points) (Supplementary Fig. 3). The 5-week washout period was selected based on the prescription duration for a medicine with a standard 28- or 30-day pack size, to allow time for a supply of a medicine dispensed immediately before the index date to end prior to follow-up. The segmented regression models compared the baseline level of medicine use at the start of the pre-intervention period, step changes (i.e., changes in level of medicine use at the start of the washout and follow-up periods) and ramp changes (i.e., gradual slope changes during the pre-intervention, washout, and follow-up periods) among those who did and did not receive an RMMR to estimate both immediate and sustained changes in medicines use over time. Complete case analysis was conducted and the statistical significance level was set at 5%.

Segmented regression models were undertaken using Stata *itsa* module [34, 35] that estimated coefficients using ordinary least squares regression with Newey-West standard errors (Stata v16.0; StataCorp, College Station, Texas). Cumby-Huizinga and Arellano-Bond tests were applied to identify autocorrelation and/or seasonality, with lag terms applied to regression models where necessary. All other analyses were undertaken using R statistical package v3.6.0 (R Foundation for Statistical Computing, Vienna, Austria).

### Supplementary analyses

Segmented regression modelling was also used to examine weekly trends in prevalence of use (i.e., the percentage of individuals receiving at least one day supply of the medicine in a week) before and after the index date among residents with an index date in the 6–12 months post-RACF entry.

## Results

There were 113909 residents from 1979 RACFs included in this study, of whom 23979 (21.1%) had an RMMR within 0–3 months after RACF entry, 19080 (16.8%) received an RMMR within 3–6 months and 11962 (10.5%) received an RMMR within 6–12 months of RACF entry (Fig. 1). A total of 58888 unexposed individuals were included (51.7% of the total cohort), of whom 55609, 49468 and 42573 individuals were assigned index dates within 0–3, 3–6 and 6–12 months after RACF entry, respectively, as eligible unexposed individuals could be assigned different index dates for each analysis period.

Overall, at RACF entry the cohort median age was 85 (interquartile range (IQR) 80–89) years, 64.1% were women, and 49.9% were living with dementia. Residents were dispensed a median of 11 (IQR 7–15) unique prescriptions for PBS-listed medicines in the year prior to RACF entry and the median comorbidity score was 5 (IQR 3–7). Resident characteristics stratified by the timing of the index date relative to RACF entry are presented in Table 1. Individuals who received an RMMR in the 0–3 months after RACF entry were more likely to reside in a major city than those who did not receive an RMMR, but geographical differences between those with and without RMMRs in the 3–6 and 6–12 months after RACF entry were minimal. Minor differences in care needs with regards to activities of daily living, behavioral daily living and complex health care were observed. Characteristics of exposed and unexposed individuals were otherwise similar regardless of the timing of the index date relative to RACF entry.

**Table 1:** Characteristics of the study cohort, stratified by the index date relative to RACF entry

Characteristic	Index date in the 0 to 3 months after RACF entry		Index date in the 3 to 6 months after RACF entry		Index date in the 6 to 12 months after RACF entry	
	**Received RMMR (*n* = 23979)**	**No RMMR (*n* = 55609)**	**Received RMMR (*n* = 19080)**	**No RMMR (*n* = 49468)**	**Received RMMR (*n* = 11962)**	**No RMMR (*n* = 42573)**
Number of RACFs	1772	1959	1809	1949	1770	1925
Age (years) at RACF entry, median (IQR)	85.0 (80.0–89.0)	85.0 (80.0–89.0)	85.0 (80.0–89.0)	85.0 (80.0–89.0)	84.0 (79.0–89.0)	85.0 (80.0–89.0)
Female, n (%)	15367 (64.1)	35233 (63.4)	12403 (65.0)	32143 (65.0)	7938 (66.4)	28324 (66.5)
Born in Australia, n (%)^a^	14798 (62.0)	36452 (65.8)	12358 (65.0)	32358 (65.7)	7911 (66.5)	27803 (65.6)
Primary language other than English, n (%)^b^	3571 (14.9)	7014 (12.6)	2447 (12.8)	6296 (12.7)	1401 (11.7)	5461 (12.8)
RACF provider type, n (%)						
For profit	10368 (43.2)	23373 (42.0)	8086 (42.4)	20525 (41.5)	4986 (41.7)	17407 (40.9)
Government	1166 (4.9)	3228 (5.8)	923 (4.8)	2743 (5.5)	710 (5.9)	2265 (5.3)
Not for profit	12445 (51.9)	29008 (52.2)	10071 (52.8)	26200 (53.0)	6266 (52.4)	22901 (53.8)
Remoteness of residence^c^, n (%)						
Major Cities	18476 (77.1)	38172 (68.6)	13551 (71.0)	34092 (68.9)	8021 (67.1)	29463 (69.2)
Outside Major Cities	5503 (22.9)	17437 (31.4)	5529 (29.0)	15376 (31.1)	3941 (32.9)	13110 (30.8)
State of residence, n (%)						
New South Wales	10963 (45.7)	26184 (47.1)	8957 (46.9)	23160 (46.8)	5225 (43.7)	19885 (46.7)
South Australia	2801 (11.7)	9028 (16.2)	2082 (10.9)	8102 (16.4)	1596 (13.3)	7065 (16.6)
Victoria	10215 (42.6)	20397 (36.7)	8041 (42.1)	18206 (36.8)	5141 (43.0)	15623 (36.7)
No. of unique prescriptions dispensed in the year before RACF entry, median (IQR)	11.0 (7.0–15.0)	11.0 (7.0–15.0)	11.0 (7.0–15.0)	11.0 (7.0–15.0)	11.0 (7.0–15.0)	11.0 (7.0–15.0)
Rx-risk comorbidity score^d^, median (IQR)	5.0 (3.0–7.0)	5.0 (3.0–7.0)	5.0 (3.0–7.0)	5.0 (3.0–7.0)	5.0 (3.0–7.0)	5.0 (3.0–7.0)
Dementia^e^, n (%)	12457 (51.9)	27534 (49.5)	9540 (50.0)	24719 (50.0)	5869 (49.1)	21344 (50.1)
Assisted Daily Living level^f^, n (%)						
Nil	308 (1.3)	1028 (1.8)	365 (1.9)	987 (2.0)	243 (2.0)	920 (2.2)
Low	6517 (27.2)	15613 (28.1)	5741 (30.1)	14697 (29.7)	3924 (32.8)	13301 (31.2)
Medium	8042 (33.5)	18841 (33.9)	6478 (34.0)	16997 (34.4)	4041 (33.8)	14722 (34.6)
High	9112 (38.0)	20127 (36.2)	6496 (34.0)	16787 (33.9)	3754 (31.4)	13630 (32.0)
Behavioral Daily Living level^f^, n (%)						
Nil	1967 (8.2)	5416 (9.7)	1739 (9.1)	4981 (10.1)	1262 (10.6)	4426 (10.4)
Low	5498 (22.9)	13249 (23.8)	4660 (24.4)	12142 (24.5)	3071 (25.7)	10716 (25.2)
Medium	6297 (26.3)	14571 (26.2)	4968 (26.0)	12853 (26.0)	3109 (26.0)	11027 (25.9)
High	10217 (42.6)	22373 (40.2)	7713 (40.4)	19492 (39.4)	4520 (37.8)	16404 (38.5)
Complex Health Care level^f^, n (%)						
Nil	1639 (6.8)	4582 (8.2)	1509 (7.9)	4314 (8.7)	1065 (8.9)	3955 (9.3)
Low	7156 (29.8)	16782 (30.2)	6036 (31.6)	15470 (31.3)	3947 (33.0)	13671 (32.1)
Medium	6255 (26.1)	14994 (27.0)	5066 (26.6)	13507 (27.3)	3236 (27.1)	11665 (27.4)
High	8929 (37.2)	19251 (34.6)	6469 (33.9)	16177 (32.7)	3714 (31.0)	13282 (31.2)
Residents remaining in the cohort at the end of 4-month follow-up, n (%)	19253 (80.3)	42398 (76.2)	15741 (82.5)	40212 (81.3)	9652 (80.7)	34576 (81.2)

*IQR* interquartile range, *RACF* residential aged care facility, *RMMR* residential medication management review

^a^ Data missing for *n* = 331 residents in the 0–3-month cohort, *n* = 276 in the 3–6-month cohort and *n* = 253 residents in the 6–12-month cohort

^b^ Data missing for *n* = 121 residents in the 0–3-month cohort, *n* = 107 in the 3–6-month cohort and *n* = 92 residents in the 6–12-month cohort

^c^ RACF remoteness (major city or other) was determined from the Australian Standard Geographical Classification [36]

^d^ Comorbidity score was derived using the Australian adaptation of 46-item Rx-Risk pharmaceutical-based comorbidity index [37]

^e^ Dementia diagnosis was determined from the diagnoses reported in the Rx-Risk, aged care eligibility and entry into care assessments [23]

^f^ Care needs with respect to activities of daily living, behavioral daily living and complex care needs (each categorized as nil, low, medium, high) were determined from data recorded during the entry into care assessments [25]

### Trends in medicines use among individuals with index dates in the 0–3 or 3–6 months after RACF entry

Considerable weekly variation in DDDs/1000 resident-days prior to the index date was observed for those with and without an RMMR for most medicines investigated (examples shown in Supplementary Figs. 4 and 5). After the index date, small decreases in statin and proton pump inhibitor (PPI) DDDs/1000 resident-days were observed among RMMR recipients compared to individuals without an RMMR in the 0–3 months after RACF entry, with fewer DDDs/1000 resident-days in the RMMR group in the last week of follow-up (statins: 457 versus 482 DDDs/1000 resident-days; PPIs: 449 versus 467 DDDs/1000 resident-days) (Supplementary Table 2, Supplementary Fig. 4). Small decreases in DDDs/1000 resident-days were also observed after an RMMR compared to those without an RMMR in the 3–6 months after RACF entry for the following medicines: stains, PPIs, benzodiazepines/zopiclone, calcium channel blockers (CCBs), and antidepressants (Supplementary Table 2, Supplementary Fig. 5).

### Trends in medicines use among individuals with an index date in the 6–12 months after RACF entry

**Fig. 2:**
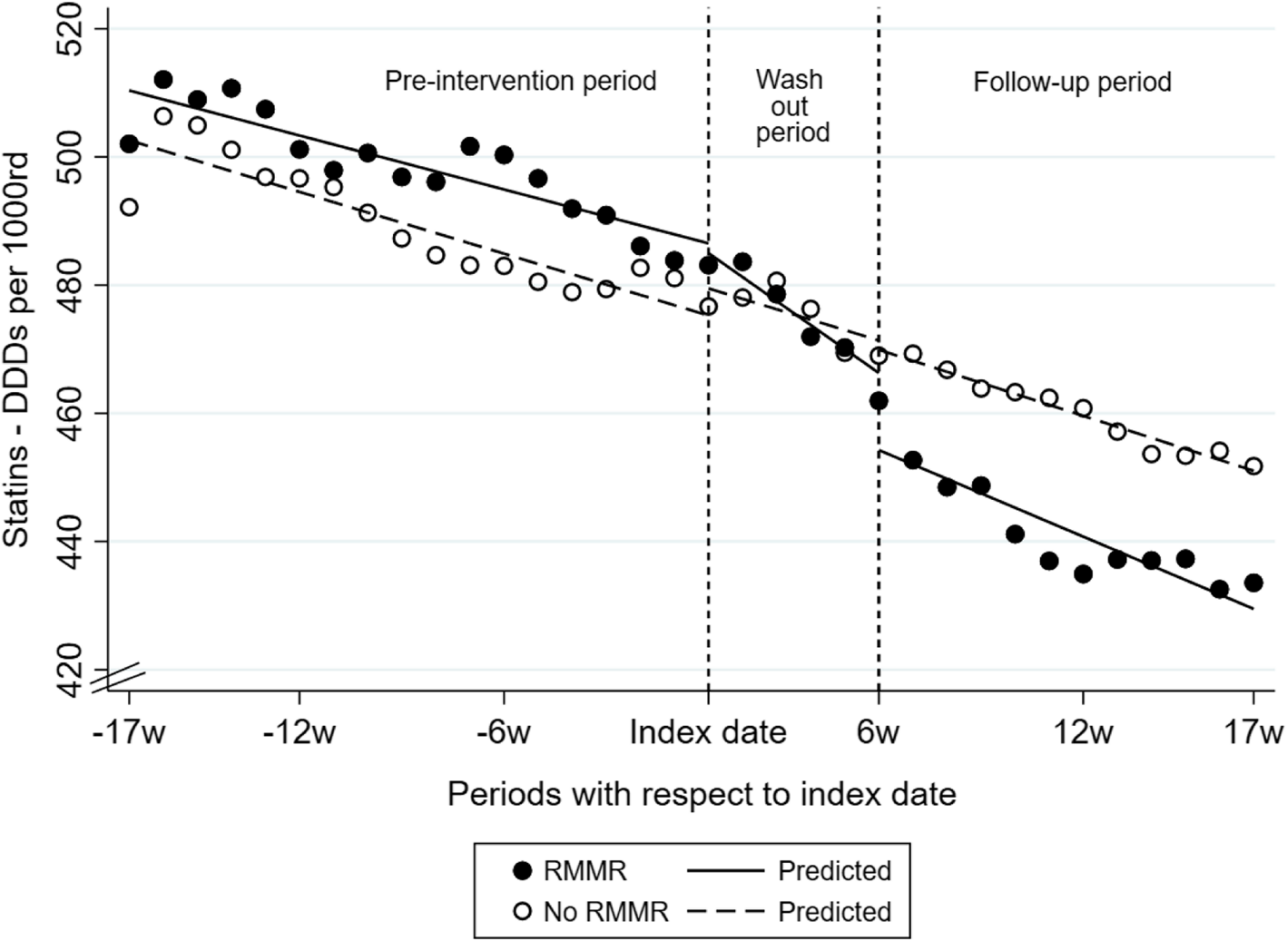
Trends in weekly number of defined daily doses of per 1000 resident-days for statins among individuals with and without an RMMR in the 6–12 months after RACF entry

Table 2 summarizes the number of DDDs of each medicine examined per 1000 resident-days during the observation period for individuals with an index date in the 6–12 months after RACF entry and the output from the segmented regression models. Table 3 shows the example of statin use, where significant reductions in use were observed in the RMMR group after the index date. Compared to those without an RMMR, statin use declined at a faster rate in the RMMR group during the washout period (*p* < 0.001) and at the start of the follow-up period (*p* < 0.001) (Table 3, Fig. 2). Similar decreases in DDDs/1000 resident-days for CCBs and PPIs were also observed (Table 2, Supplementary Fig. 6). In addition, antidepressant use plateaued in the RMMR group after the index date but continued to slowly increase in those without an RMMR (*p* < 0.001). Small changes in benzodiazepine and antipsychotic use also occurred post-RMMR (0.4 DDDs/1000 resident-days/week). For all other medicines, there were either no significant differences in trends for those who did and did not receive an RMMR, or outlier values at the start or end of follow-up impacted the trends observed (Table 2).

**Table 2:** Weekly number of defined daily doses of medicines per 1000 resident-days during the study period and summary of trends in individuals who did and did not receive an RMMR in the 6–12 months after RACF entry

Medicine class	RMMR exposure status	Weekly DDDs/1000 resident-days			Summary of trends
		**17-weeks before index date**	**Week of index date**	**17-weeks after index date**	
Antidepressants^a^	RMMR	459.3	491.7	488.0	• During the pre-intervention period and washout periods, antidepressant use was increasing at a similar rate in both groups • During follow-up, use plateaued in the RMMR group, but continued to slowly increase in those without an RMMR (-0.34 vs. 0.66 DDDs/1000 days per week, *p* < 0.001)
	No RMMR	434.5	460.5	468.7	
Antipsychotics	RMMR	67.0	67.4	67.0	• Differences in weekly trends between groups during the pre-intervention and follow-up periods were negligible (≤ 0.1 DDDs/1000 days per week)
	No RMMR	64.1	65.8	66.6	
Benzodiazepines or zopiclone	RMMR	102.2	106.0	99.9	• During the pre-intervention period, benzodiazepine/zopiclone use increased at a similar rate in both groups (*p* = 0.524) • Use declined more quickly in the RMMR group during the washout and follow-up periods, but the differences in trends were negligible (0.4 DDDs/1000 days per week, *p* = 0.001)
	No RMMR	96.7	100.1	99.6	
Opioids	RMMR	87.5	101.0	101.5	• During the pre-intervention period, opioid use increased in those with and without an RMMR, with only a negligible difference between groups of 0.1 DDDs/1000 days per week • Opioid use plateaued after the index date with no difference in trends between the two groups (*p* > 0.05)
	No RMMR	86.6	96.9	97.3	
Medicines for cognitive symptoms of dementia	RMMR	119.0	113.8	112.4	• There were no significant differences in use between the two groups during the pre-intevention and washout periods (all *p* > 0.05) • A small difference in weekly use between groups during the follow-up period (-0.7 vs. -0.4 DDDs/1000 days per week, *p* = 0.03) was observed. Cautious interpretation is advised due to considerable variation in use (weekly differences of up to 10 DDDs/1000 days) observed in the RMMR group during follow-up
	No RMMR	116.2	112.6	111.4	
Proton pump inhibitors^a^	RMMR	489.0	499.5	466.2	• During the pre-intervention period, PPI use was increasing at a similar rate in both groups (*p* = 0.596) • During the washout period, PPI use declined faster in the RMMR group (-2.6 vs. -1.5 DDDs/1000 days per week, *p* = 0.001) • At the start of the follow-up period, the rate of PPI use in the RMMR group dropped below the rate among those without an RMMR (-14.0 vs. -2.8 DDDs/1000 days, *p* < 0.001) and continued to decline at the same rate in both groups thereafter (*p* = 0.334)
	No RMMR	477.9	488.7	474.3	
Osteoporosis medicines	RMMR	151.1	152.9	151.6	• There were no significant differences in use between the two groups during the pre-intevention, washout and follow-up periods (all *p* > 0.05) • In the first week of the follow-up period, a significant drop in use was observed in the RMMR group (-3.0 vs. 0.04 DDDs/1000 days, *p* = 0.001). However, this was influenced by an outlier observation at the end of the follow-up period
	No RMMR	150.6	147.1	145.8	
Glucose lowering medicines	RMMR	282.2	262.4	249.7	• Use decreased at a similar rate in both groups during the pre-intervention, washout and follow-up periods (all *p* > 0.05)
	No RMMR	292.9	261.5	251.3	
Statins^a^	RMMR	502.1	483.1	433.6	• Significant decrease in statin use in the RMMR group post-index date; refer to Table 3 and description in “Results“ section
	No RMMR	492.2	476.7	451.8	
ACE inhibitors or sartans	RMMR	636.4	628.4	608.2	• ACE inhibitor/sartan use decreased at a similar rate in both groups during the pre-intervention and follow-up periods (*p* > 0.05) • There was a slightly faster rate of decline in the RMMR group during the washout period (-3.7 vs -1.6 DDDs/1000 days per week, *p* = 0.01). However, this was influenced by an outlier observation in the first week of the washout period
	No RMMR	609.2	601.7	597.5	
Beta blockers	RMMR	137.8	131.8	125.0	• Differences in weekly trends between groups during the pre-intervention, washout and follow-up periods were negligible (≤ 0.4 DDDs/1000 days per week)
	No RMMR	130.3	132.5	124.8	
Calcium channel blockers (CCBs)^a^	RMMR	241.6	243.3	228.0	• CCB use decreased at a similar rate in both groups during the pre-intervention and washout periods (*p* = 0.435) • At the start of the follow-up period, CCB in the RMMR group dropped below the rate in the group without an RMMR (-4.2 versus 2.1 DDDs/1000 days, *p* < 0.001), but declined more quickly among those without an RMMR during follow-up (-0.12 vs. -0.62 DDDs/1000 days per week, *p* < 0.001)
	No RMMR	241.2	240.6	232.3	
Loop diuretics	RMMR	376.4	395.8	385.2	• During the pre-intervention period, use was increasing at a slightly faster rate among individuals RMMR (0.92 vs. 1.8 DDDs/1000 days per week, *p* = 0.015). However, use declined during the washout and follow-up periods, with no differences in trends observed between the two groups (*p* > 0.05)
	No RMMR	355.9	386.7	366.6	
Oral anticoagulants	RMMR	74.8	74.1	68.0	• Use was stable during the pre-intervention period, with only a negligible difference between groups (< 0.1 DDDs/1000 days per week) • Oral anticoagulant use declined at a similar rate in both groups after the index date (*p* > 0.05)
	No RMMR	72.0	71.9	65.6	

*ACE* Angiotensin converting enzyme, *CCB* calcium channel blocker, *DDD* Defined daily dose, *RACF* Residential aged care facility, *RMMR* Residential Medication Management Review

^a^ Medicine classess with significant changes in weekly DDDs/1000 resident-days after the index date in the RMMR group compared to those without an RMMR

**Table 3:** Segmented regression output showing impact of RMMR on weekly statin DDDs per 1000 resident-days during pre-intervention, washout, and follow-up periods for individuals with an index date in the 6–12 months after RACF entry

	**Received RMMR (exposed)**		**Did not receive an RMMR (unexposed)**		**Difference between exposed and unexposed**	
	**Estimate (95% CI)**	***p* -value**	**Estimate (95% CI)**	***p* -value**	**Estimate (95% CI)**	***p *-value**
Use at baseline^a^	510.4 (508.4 to 512.4)	< 0.001	502.6 (500.3 to 504.9)	< 0.001	7.8 (4.7 to 10.8)	< 0.001
Pre-intervention trend^b^	-1.4 (-1.6 to -1.2)	< 0.001	-1.6 (-1.9 to -1.4)	< 0.001	0.21 (-0.14 to 0.55)	0.240
Change at start of washout^c^	-1.5 (-4.0 to 1.0)	0.225	4.2 (1.3 to 7.1)	0.005	-5.7 (-9.6 to -1.9)	0.004
Trend during washout period^d^	-3.7 (-4.0 to -3.5)	< 0.001	-1.6 (-2.4 to -1.3)	< 0.001	-2.1 (-3.0 to -1.3)	< 0.001
Change at start of follow-up^e^	-12.0 (-15.7 to -8.4)	< 0.001	-1.5 (-4.0 to 1.1)	0.257	-10.6 (-15.0 to -6.1)	< 0.001
Follow-up trend^f^	-2.3 (-2.8 to -1.7)	< 0.001	-1.7 (-1.8 to -1.7)	< 0.001	-0.54 (-1.12 to 0.05)	0.072

^a^ Number of statin DDDs available for use per 1000 resident-days in the first week of the study (-17w) (values are predicted from the regression model)

^b^ The slope of the linear regression line (i.e., the weekly rate of change in statin DDDs/1000 days) in the pre-intervention period

^c^ The immediate step/change in statin use in the first week of the washout period (i.e., the first week after the index date)

^d^ The slope of the linear regression line (i.e., the weekly rate of change in statin DDDs/1000 days) in the washout period

^e^ The immediate step/change in statin use in the first week of the follow-up period

^f^ The slope of the linear regression line (i.e., the weekly rate of change in statin DDDs/1000 days) in the follow-up period

*CI* confidence interval, *DDD* defined daily dose, *RACF* residential aged care facility, *RMMR* Residential Medication Management Review

### Supplementary analysis

Supplementary Table 3 summarizes prevalence of use of each medicine class for individuals with an index date in the 6–12 months after RACF entry. Decreases in prevalence of use of statins, benzodiazepines/zopiclone, CCBs, PPIs and antipsychotics were observed post-RMMR in comparison to those without an RMMR (Supplementary Fig. 7). The prevalence of oral anticoagulant use decreased at a slower rate in the washout and follow-up periods among those with an RMMR compared to those who did not receive an RMMR.

## Discussion

This study of 113909 residents from 1979 facilities, capturing 67% of all older Australians with a new RACF admission nationally over a four-year period [38], is the first examination of population-based trends in medicines use following provision of an RMMR in the 25-year history of Australia’s RMMR program. Decreased use of statins and PPIs were observed across all three time periods examined, and small decreases in CCBs, benzodiazepines/zopiclone, and antidepressants post-RMMR were observed following provision of an RMMR in the 3–6 and 6–12 months after RACF entry by comparison to those without RMMRs. These changes, although modest, may translate to meaningful differences at the population level due to high exposure to these medicines in RACFs. For example, extrapolation of the prevalence estimates for statins among individuals with an index date in the 6–12 months after RACF entry to all 60,723 new entrants to Australian RACFs between July 2019 and June 2020 [38] suggests if all individuals received an RMMR, an estimated 486 fewer residents would be taking a statin four months later, provided all were alive at follow-up. This finding likely reflects an increasing focus on statin discontinuation among individuals with limited life expectancy or severe cognitive or functional decline during the analysis period [39].

Although RMMRs are generally recommended for all individuals on RACF entry and when clinical circumstances change [9, 23], changes in use of a greater number of medicines were observed among individuals receiving an RMMR in the 6 to 12 months after entering an RACF. This is despite similarities in both resident characteristics and DDDs/1000 resident-days for individual medicine classes across all three periods. It may be that RMMRs conducted on RACF entry have a different focus to those conducted when an individual is more settled in the RACF. Numerous activities undertaken during RMMRs could enhance medicines safety and effectiveness or resident quality of life without directly impacting trends in medicines use, including: medicines reconciliation, assessment of an individual’s ability to self-administer medicines, medicines simplification, identifying non-pharmacological approaches, screening for adverse drug events, recommending pathology tests and health professional referrals, and providing education [9, 13]. Pharmacists likely spend more time on activities such as medicines reconciliation for new residents due to known problems with information transfer during care transitions and because there are often changes to resident’s usual GP on RACF entry [40–42]. For long-term residents with an established history of medicines use, there may be greater opportunity to focus on other areas such as deprescribing. Additionally, GPs may be hesitant to deprescribe psychotropics immediately on RACF entry for residents whose admissions are preceded by worsening behavioral and psychological symptoms of dementia and may prefer to delay deprescribing attempts until a resident is settled in the RACF. Our findings suggest further research into optimal timepoints for delivering services to enhance medicines use and resident health outcomes could be beneficial, particularly given recent changes to the RMMR program that enable pharmacists to provide up to two follow-up visits and the increasing interest in integrating pharmacists within RACFs to deliver clinical services [43]. Future directions also include the need to characterize pharmacist activities and associated quality and safety measures across a resident’s entire RACF journey to ensure medicines management needs are met and identify areas for improvement.

Another factor impacting the opportunity to observe changes in medicines use post-RMMR was the considerable variability in use before the index date for residents with index dates 0–3 and 3–6 months post-RACF entry. Increases in both prevalence of use and frequency of dispensing due to supply arrangements at hospital discharge and RACF entry likely contributed to this variability. Recent studies report increased use of psychotropics, laxatives and medicines for dementia in the year before and on entry to an RACF [3, 30, 44]. Medicines are often re-dispensed at RACF entry and packed into dose administration aids, with any existing supplies discarded, possibly resulting in an increased number of DDDs available for use. However, trends in medicines use over the four months before the index date had generally stabilized for individuals with an index date 6–12 months post-RACF entry, which may have made it easier to observe medicines changes in this cohort. Hence, careful selection of study cohorts and methodological approaches for studies exploring the impact of interventions delivered in RACFs to optimize medicines use after RACF entry are needed.

The modest reductions in weekly trends in statin, PPI, and CCB use in the four months after an RMMR suggest dose reductions, switches to *pro re nata* (when required) use (for PPIs) and/or deprescribing occurs more frequently after an RMMR than initiation or dose increases. This is in line with existing guidance that suggests statins, PPIs and some antihypertensives could be targeted for deprescribing in RACFs in certain circumstances [45–48]. Although we were unable to ascertain the appropriateness of medicines use in this study, population-based reductions in the use of these medicines could have important implications for both patients and healthcare funders. This includes potential reductions in the cost of medicines and unnecessary harm due to adverse drug events (e.g., *Clostridioides difficile* infection associated with PPI use).

However, during the period of this analysis, RMMRs had minimal impact on psychotropic medicines use at the population-level. This is despite long-standing concerns regarding an overreliance on antipsychotics and benzodiazepines in RACFs in Australia and internationally [1]. This concern has heightened since the analysis period, particularly during the recent Royal Commission into Aged Care Quality and Safety in Australia, and future research should analyze the effect of RMMRs on psychotropic medicines in the period after the Commissioner’s interim recommendations [15]. Several factors may have contributed to an absence of population-based changes in other medicines post-RMMR compared to those without RMMRs, including known barriers to deprescribing [49] and RMMR delivery [23, 50, 51] in RACFs. Our study examined trends medicines use in the four months post-RMMR however it is possible that the deprescribing may be implemented over a longer period as some medicines may need to be discontinued slowly, and generally only one to three medicines can be deprescribed concurrently [48]. The impact of an RMMR and any resulting population-level changes in medicines use may also be impacted by the quality and type of recommendations made by pharmacists during an RMMR, GP attitude, beliefs, and experiences with deprescribing, opportunities for shared decision-making, interprofessional relationships and communication, and varying rates of GP implementation of recommendations. The top three recommendations made by pharmacists during RMMRs comprise identifying the need for clinical or laboratory monitoring (27% of recommendations), dose/schedule changes (21.4%) and ceasing or withdrawing a medicine (16%) [13]. Although 45–84% of pharmacists’ RMMR recommendations are accepted by GPs [13], uptake varies by recommendation type. GPs accept two thirds (64%) of recommendations focusing on medicines cessation, while 85–98% of recommendations not directly involving changes to medicines (i.e., referrals to other health professionals, pathology testing or education) are accepted by GPs [13]. Together, these findings indicate RMMR program changes and other system changes are needed to address known barriers, enhance service delivery, support interprofessional collaboration and incorporate new models of care [52] to support deprescribing in RACFs.

### Strengths and limitations

This study utilized a population-based approach to examine short-term trends in medicines use after an RMMR. Study strengths include the use of Australia’s largest and most comprehensive linked health dataset of older people accessing RACF care to examine trends in medicines that are commonly utilized in RACFs. Use of CITS rather than single group interrupted time series enabled comparison of trends among those with and without an RMMR in the 6–12 months after RACF entry [53, 54]. Examination of medicines use over four-months either side of the index date limited the risk of time-varying confounding due to other interventions during the study period or changes in an individual’s clinical circumstances [54]. DDDs/1000 days is a standardized, widely used measure of drug utilization although doses prescribed to older residents can be different to the DDD for medicines such as opioids [55] and antipsychotics. Importantly, supplementary analyses examining trends in prevalence of use showed patterns consistent with the main analysis.

Our analysis used GP MBS claims to determine RMMR provision. Pharmacists claim for RMMRs via a different mechanism that is not captured in our dataset. Hence, study limitations include the possible misclassification of RMMR exposure as fewer claims for RMMRs are submitted by GPs compared to pharmacists [23, 29]. However, the impact of any misclassification would be towards the null (i.e., no difference between trends, thereby underestimating the impact of an RMMR on medicines use). This may in part explain why changes in medicines use were sometimes observed after the index date for those classified as not receiving an RMMR. Other limitations are that we could not examine specific recommendations made during the RMMR or appropriateness of medicines use. Linkage between ROSA and the pharmacist RMMR claims dataset could help to address some of these knowledge gaps in the future.

Other limitations were that we could not ascertain use of non-government subsidized medicines, although any resulting impact is likely to be minor as most medicines are PBS-listed and 97% of residents of RACFs hold a government-issued concession card that entitles them to subsidized medicines at the lowest copayment level (AUD $6.80 in 2022) [56]. Overall, the medicines examined in this study comprised 502901 of the 856492 (58.7%) prescriptions dispensed over a 12-month period for all 11792 individuals with an index date in the final year of our study (2016). Hence, while we examined the most utilized medicines, including four classes of high-risk medicines prioritized for national action to reduce severe avoidable medicines-related harm [14], we did not examine use of medicines such as urinary anticholinergics, digoxin, or medicines for Parkinson’s disease. Medicines for residents are often provided in dose administration aids which may impact dispensing patterns. Additionally, medicines use may have been overestimated as not all medicines dispensed are necessarily administered, with impact greatest for medicines with long prescription durations. For example, insulin glargine has a prescription duration of 210 days (30 weeks), which means individuals with a dispensing in the 13 weeks prior to the index date would be considered as taking the medicine for the remainder of the observation period. We examined changes in specific medicines at the population level but did not examine switching between classes or changes in overall exposure to potentially inappropriate medicines or those contributing to anticholinergic and/or sedative burden. Recent changes to RMMR program rules in early 2020 that enable up to two pharmacist follow-up visits and temporary delivery via telehealth [51] require further evaluation.

## Conclusions

This population-based examination of medicines use found modest changes in use of statins, PPIs, CCBs, antidepressants, and small changes in use of benzodiazepines/zopiclone and antipsychotics following provision of an RMMR in the 6–12 months after RACF entry compared to those who did not receive an RMMR. No population-based changes were observed after an RMMR for the eight other medicine classes examined despite high rates of exposure to these medicines in RACFs. Our findings have important implications for care recipients, providers, and policy makers. Although RMMRs are recommended for all residents on entry to an RACF, medicines use is highly variable during this period. Our findings suggest that activities such as medicines reconciliation may be prioritized when an RMMR is provided on RACF entry, with deprescribing more likely to occur after an RMMR the longer a resident has been in the RACF. Although deprescribing is not the primary goal of an RMMR, the modest changes in psychotropic medicines use when an RMMR is provided in the 6–12 months after RACF entry suggest enhancements to the Australian medicines review program in RACFs and other systems changes are required to effectively address the high rates of psychotropic medicines use in RACFs. Determining associations between RMMR provision and health outcomes such as hospitalization remains an important future research direction.

## Supplementary Information

**Additional file 1: Supplementary Table 1**. Codes used to identify medicine classes of interest, and corresponding prescription durations. **Supplementary Table 2**. Weekly number of defined daily doses of medicines per 1000 resident-days before and after the index date among individuals who did and did not receive an RMMR in the 0-3 months and 3-6 months after RACF entry. **Supplementary Table 3**. Weekly prevalence of medicines use during the study period among individuals with an index date in the 6-12 months after RACF entry. **Supplementary Figure 1**. Group assignment based on the date of an individual’s first RMMR relative to first entry into permanent residential aged care. **Supplementary Figure 2**. Time frame for assessing medicines use pre- and post-index date. **Supplementary Figure 3**. Time periods analyzed in the segmented regression models. **Supplementary Figure 4.** Weekly number of DDDs available for use per 1000 resident-days for individuals with an index date in the 0-3 months after RACF entry for medicines with possible changes in use post-RMMR compared to individuals without an RMMR. **Supplementary Figure 5.** Weekly number of DDDs available for use per 1000 resident-days for individuals with an index date in the 3-6 months after RACF entry for medicines with possible changes in use post-RMMR compared to individuals without an RMMR. **Supplementary Figure 6**. Weekly number of DDDs per 1000 resident-days in the four months before and after the index date for individuals with an index date within 6-12 months of RACF entry. **Supplementary Figure 7**. Weekly prevalence of medicines use in the four months before and after the index date for individuals with an index date in the 6-12 months after RACF entry.

## Data Availability

The data for this study were obtained from the Australian Institute of Health and Welfare and Australian Government Department of Health. These data were made available to the researchers under ethical, governance, and confidentiality agreements that do not allow public sharing. JKS can be contacted to discuss data and materials utilized for this study.

## Abbreviations

AACP: Australian Association of Consultant Pharmacy
ACE: Angiotensin Converting Enzyme
ATC: Anatomical Therapeutic Chemical
CCB: Calcium channel blocker
CI: Confidence interval
CITS: Controlled interrupted time series
DDD: Defined daily dose
GP: General medical practitioner
HMR: Home Medicines Review
IQR: Interquartile range
MBS: Medicare Benefits Schedule
NHMRC: National Health and Medical Research Council
NSW: New South Wales
PBS: Pharmaceutical Benefits Scheme
PPI: Proton pump inhibitor
RACF: Residential aged care facility
ROSA: Registry of Senior Australians
RMMR: Residential Medication Management Review
SA: South Australia
VIC: Victoria
WHO: World Health Organization
