# Supplementary material for: Medicines use before and after comprehensive medicines review among residents of long-term care facilities: a retrospective cohort study
Source: BMC Geriatr. 2022 Jun 8;22:493. doi: 10.1186/s12877-022-03187-0 (PMC9178815; doi:10.1186/s12877-022-03187-0)

**Supplementary Table 1**. Codes used to identify medicine classes of interest, and corresponding prescription durations

| **Medicine class** | **WHO ATC code** | **Prescription duration* (days), [range]** |
| --- | --- | --- |
| Antidepressants | N06A | 33 – 57 |
| Antipsychotics | N05A, excluding prochlorperazine (N05AB04) and lithium (N05AN) | 23 – 144 |
| Benzodiazepines and/or zopiclone | N05BA, N05CD, N05CF, N03AE | 6 – 135 |
| Opioids | N02A | Determined based on quantity dispensed |
| Medicines for cognitive symptoms of dementia | N06D | 32 – 68 |
| Proton pump inhibitors | A02BC | 35 – 39 |
| Osteoporosis medicines | A11CC04, G03XC01, H05AA02, M05BA01, M05BA04, M05BA05, M05BA07, M05BB01 to M05BB07, M05BX03, M05BX53; and PBS item codes 9350D, 9288W, 10555M, 10571J, 5457F | 32 – 365 |
| Glucose lowering medicines | A10 | 31 – 210 |
| Statins | C10AA, C10B, A10BH51 | 33 – 36 |
| ACE inhibitors and/or sartans | C09A, C09B, C09C, C09D | 29 – 75 |
| Beta blockers | C07 | 32 – 98 |
| Calcium channel blockers | C08, C09BB, C09BX01, C09BX03, C09BX04, C09DB, C09DX01, C09DX03, C09DX06, C09DX07, C10BX03 | 32 – 94 |
| Loop diuretics | C03C | 5 – 120 |
| Oral anticoagulants | B01AA, B01AE07, B01AF | 14 – 79 |

ACE Angiotensin Converting Enzyme; ATC Anatomical Therapeutic Chemical; PBS Pharmaceutical Benefits Scheme; WHO World Health Organization.

*The prescription duration for each medicine was determined from PBS claims data in 2016 and represents the number of days in which 75% of individuals receive a subsequent dispensing of the same medicine.

**Supplementary Table 2**. Weekly number of defined daily doses of medicines per 1000 resident-days before and after the index date among individuals who did and did not receive an RMMR in the 0-3 months and 3-6 months after RACF entry

| **Medicine class** | **Timing of index date relative to RACF entry** | **RMMR exposure status** | **Weekly DDDs/1000 resident-days** | | |
| --- | --- | --- | --- | --- | --- |
|  |  |  | **17-weeks before index date** | **Week of index date** | **17-weeks after index date** |
| Antidepressants | 0-3 months | RMMR | 338.4 | 477.2 | 472.3 |
|  |  | No RMMR | 317.2 | 427.6 | 435.6 |
|  | 3-6 months | RMMR | 424.4 | 470.8 | 476.6 |
|  |  | No RMMR | 418.6 | 432.7 | 455.9 |
| Antipsychotics | 0-3 months | RMMR | 36.7 | 69.7 | 64.2 |
|  |  | No RMMR | 35.2 | 71.0 | 65.6 |
|  | 3-6 months | RMMR | 57.7 | 65.2 | 67.5 |
|  |  | No RMMR | 59.0 | 66.5 | 65.8 |
| Benzodiazepines or zopiclone | 0-3 months | RMMR | 66.3 | 109.1 | 97.0 |
|  |  | No RMMR | 64.0 | 111.2 | 96.5 |
|  | 3-6 months | RMMR | 94.2 | 104.6 | 98.7 |
|  |  | No RMMR | 93.9 | 99.4 | 98.5 |
| Opioids | 0-3 months | RMMR | 63.0 | 90.7 | 88.2 |
|  |  | No RMMR | 68.6 | 96.5 | 95.5 |
|  | 3-6 months | RMMR | 78.7 | 92.6 | 96.8 |
|  |  | No RMMR | 89.6 | 96.2 | 95.4 |
| Medicines for cognitive symptoms of dementia | 0-3 months | RMMR | 115.6 | 126.3 | 119.7 |
|  |  | No RMMR | 107.3 | 113.3 | 113.4 |
|  | 3-6 months | RMMR | 127.0 | 118.8 | 122.4 |
|  |  | No RMMR | 123.3 | 112.5 | 116.1 |
| Proton pump inhibitors | 0-3 months | RMMR | 395.1 | 533.1 | 449.2 |
|  |  | No RMMR | 398.9 | 513.4 | 466.7 |
|  | 3-6 months | RMMR | 495.3 | 498.9 | 463.2 |
|  |  | No RMMR | 527.3 | 486.2 | 473.4 |
| Osteoporosis medicines | 0-3 months | RMMR | 139.2 | 161.0 | 148.9 |
|  |  | No RMMR | 137.9 | 152.4 | 142.9 |
|  | 3-6 months | RMMR | 155.8 | 154.6 | 146.5 |
|  |  | No RMMR | 156.5 | 150.6 | 146.7 |
| Glucose lowering medicines | 0-3 months | RMMR | 266.7 | 342.5 | 266.3 |
|  |  | No RMMR | 263.9 | 333.3 | 254.2 |
|  | 3-6 months | RMMR | 317.8 | 304.3 | 259.1 |
|  |  | No RMMR | 339.2 | 301.8 | 250.7 |
| Statins | 0-3 months | RMMR | 525.0 | 552.7 | 456.5 |
|  |  | No RMMR | 506.7 | 526.9 | 482.2 |
|  | 3-6 months | RMMR | 552.3 | 508.5 | 450.0 |
|  |  | No RMMR | 582.0 | 495.0 | 471.7 |
| ACE inhibitors or sartans | 0-3 months | RMMR | 679.8 | 652.5 | 608.6 |
|  |  | No RMMR | 670.8 | 621.7 | 603.0 |
|  | 3-6 months | RMMR | 682.4 | 618.9 | 602.5 |
|  |  | No RMMR | 703.0 | 598.2 | 600.9 |
| Beta blockers | 0-3 months | RMMR | 136.9 | 159.8 | 123.5 |
|  |  | No RMMR | 137.4 | 156.0 | 126.4 |
|  | 3-6 months | RMMR | 159.3 | 135.6 | 129.3 |
|  |  | No RMMR | 169.1 | 131.4 | 129.2 |
| Calcium channel blockers | 0-3 months | RMMR | 252.6 | 263.9 | 235.5 |
|  |  | No RMMR | 243.6 | 246.8 | 235.8 |
|  | 3-6 months | RMMR | 260.3 | 242.8 | 229.3 |
|  |  | No RMMR | 274.5 | 238.7 | 238.5 |
| Loop diuretics | 0-3 months | RMMR | 281.7 | 428.2 | 353.5 |
|  |  | No RMMR | 292.5 | 432.8 | 355.8 |
|  | 3-6 months | RMMR | 363.3 | 392.4 | 368.7 |
|  |  | No RMMR | 394.7 | 381.4 | 365.7 |
| Oral anticoagulants | 0-3 months | RMMR | 70.2 | 90.3 | 75.6 |
|  |  | No RMMR | 66.0 | 83.1 | 68.6 |
|  | 3-6 months | RMMR | 86.3 | 79.7 | 74.4 |
|  |  | No RMMR | 82.9 | 72.5 | 69.6 |

ACE Angiotensin converting enzyme; RACF Residential aged care facility; RMMR Residential Medication Management Review.

**Supplementary Table 3**. Weekly prevalence of medicines use during the study period among individuals with an index date in the 6-12 months after RACF entry

| **Medicine class** | **RMMR exposure status** | **Weekly prevalence of use (%)** | | |
| --- | --- | --- | --- | --- |
|  |  | **17-weeks before index date** | **Week of index date** | **17-weeks after index date** |
| Antidepressants | RMMR | 38.0 | 40.3 | 40.8 |
|  | No RMMR | 36.8 | 38.6 | 39.5 |
| Antipsychotics | RMMR | 18.9 | 19.6 | 19.3 |
|  | No RMMR | 18.6 | 19.3 | 19.6 |
| Benzodiazepines or zopiclone | RMMR | 20.7 | 21.1 | 20.4 |
|  | No RMMR | 19.6 | 20.1 | 20.0 |
| Opioids | RMMR | 21.0 | 23.6 | 24.0 |
|  | No RMMR | 20.4 | 22.6 | 23.0 |
| Medicines for cognitive symptoms of dementia | RMMR | 10.4 | 10.0 | 9.8 |
|  | No RMMR | 10.4 | 9.8 | 10.0 |
| Proton pump inhibitors | RMMR | 43.0 | 44.0 | 42.7 |
|  | No RMMR | 42.0 | 42.8 | 42.2 |
| Osteoporosis medicines | RMMR | 14.7 | 14.8 | 14.7 |
|  | No RMMR | 14.9 | 14.3 | 14.3 |
| Glucose lowering medicines | RMMR | 14.3 | 14.2 | 13.7 |
|  | No RMMR | 14.9 | 14.4 | 14.1 |
| Statins | RMMR | 35.3 | 33.4 | 30.6 |
|  | No RMMR | 35.3 | 33.3 | 32.1 |
| ACE inhibitors or sartans | RMMR | 40.8 | 40.0 | 39.5 |
|  | No RMMR | 39.5 | 38.4 | 38.4 |
| Beta blockers | RMMR | 24.0 | 24.0 | 22.7 |
|  | No RMMR | 22.7 | 23.5 | 22.4 |
| Calcium channel blockers | RMMR | 19.1 | 18.8 | 17.8 |
|  | No RMMR | 19.2 | 18.7 | 18.4 |
| Loop diuretics | RMMR | 27.2 | 28.8 | 28.8 |
|  | No RMMR | 26.4 | 28.4 | 28.0 |
| Oral anticoagulants | RMMR | 12.5 | 12.6 | 12.0 |
|  | No RMMR | 12.6 | 12.4 | 11.7 |

ACE Angiotensin converting enzyme; RACF Residential aged care facility; RMMR Residential Medication Management Review.

**Supplementary Figure 1**. Group assignment based on the date of an individual’s first RMMR relative to first entry into permanent residential aged care

**365 days post-entry**

**90 days post-entry**

**Person enters permanent care**

**Group 1**: First RMMR provided within 0 to 3 months


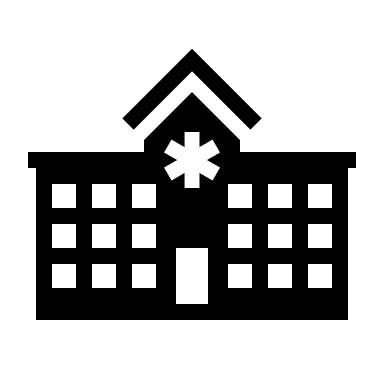


**182 days post-entry**

**Group 2**:

First RMMR provided within 3 to 6 months

**Group 3**:

First RMMR provided within 6 to 12 months


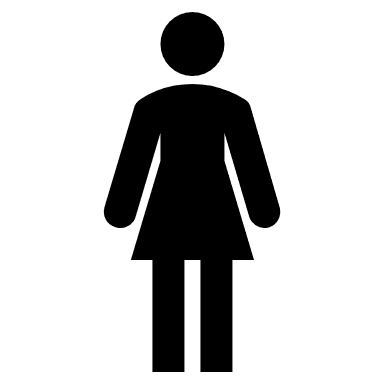


**Supplementary Figure 2**. Time frame for assessing medicines use pre- and post-index date

Lookback period for medicines supply begins here

17 weeks post-index date

17 weeks before index date

1 year before index date

**INDEX DATE**

(i.e., date of RMMR or index date assigned to comparison group)

DDDs/1000 days calculated weekly

DDDs/1000 days calculated weekly

**Supplementary Figure 3**. Time periods analyzed in the segmented regression models

5 weeks

post-index date

Washout period

(5 time points)

Follow-up period

(12 time points)

Pre-intervention period

(17 time points)

17 weeks

before index date

**INDEX**

**DATE**

17 weeks

post-index date

**Supplementary Figure 4.** Weekly number of DDDs available for use per 1000 resident-days for individuals with an index date in the 0-3 months after RACF entry for medicines with possible changes in use post-RMMR compared to individuals without an RMMR

1. Statins


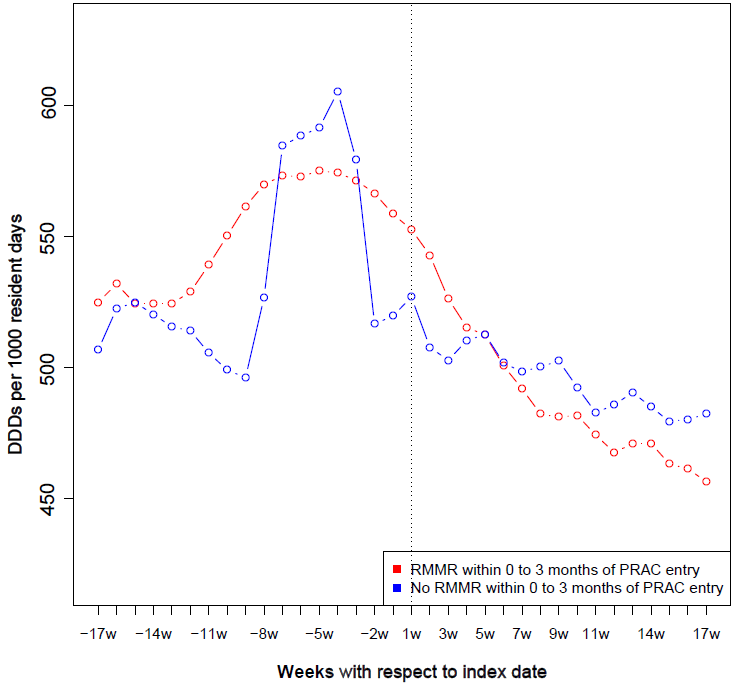


1. Proton pump inhibitors (PPIs)


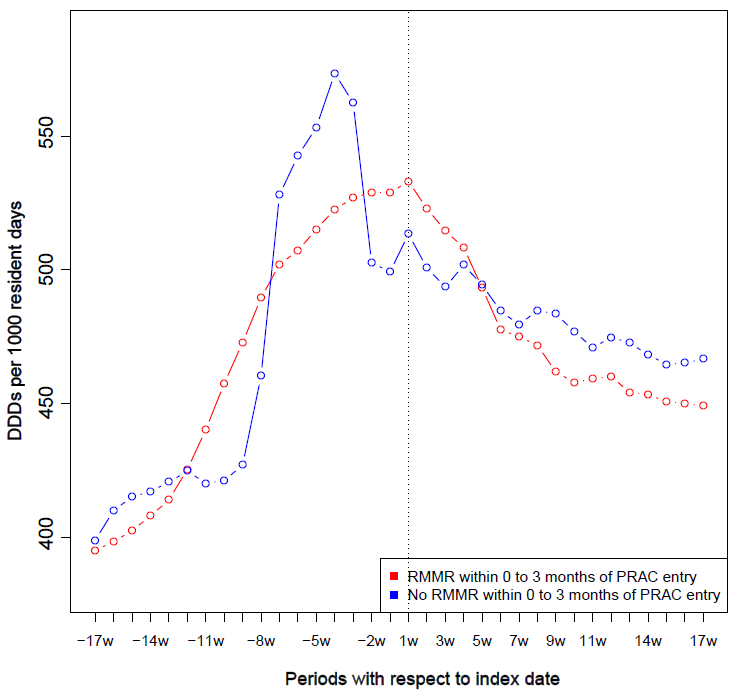


**Supplementary Figure 5.** Weekly number of DDDs available for use per 1000 resident-days for individuals with an index date in the 3-6 months after RACF entry for medicines with possible changes in use post-RMMR compared to individuals without an RMMR

1. Statins


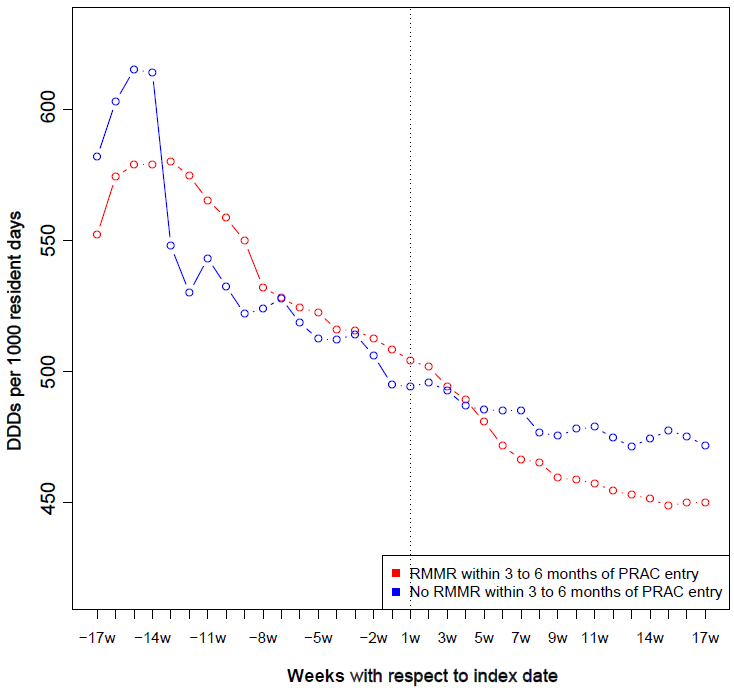


1. Proton pump inhibitors (PPIs)


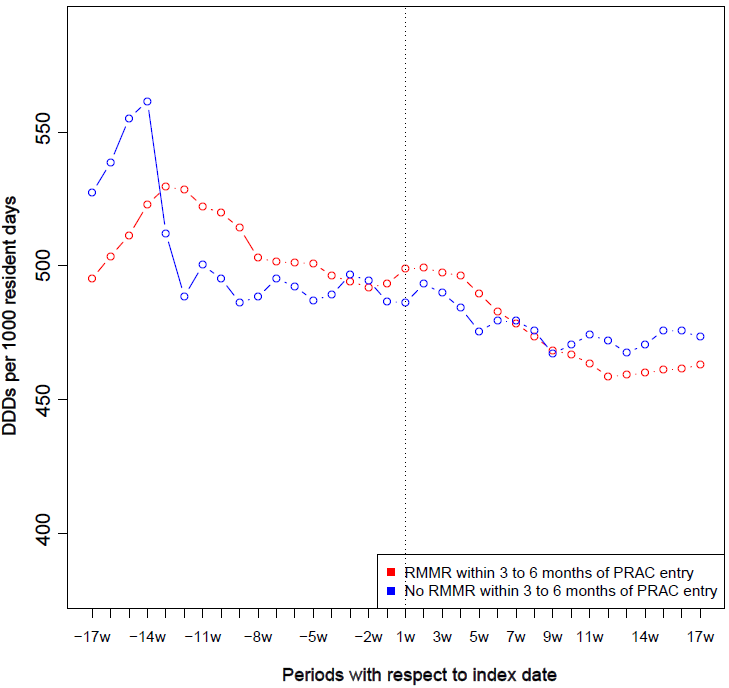


1. Calcium channel blockers (CCBs)


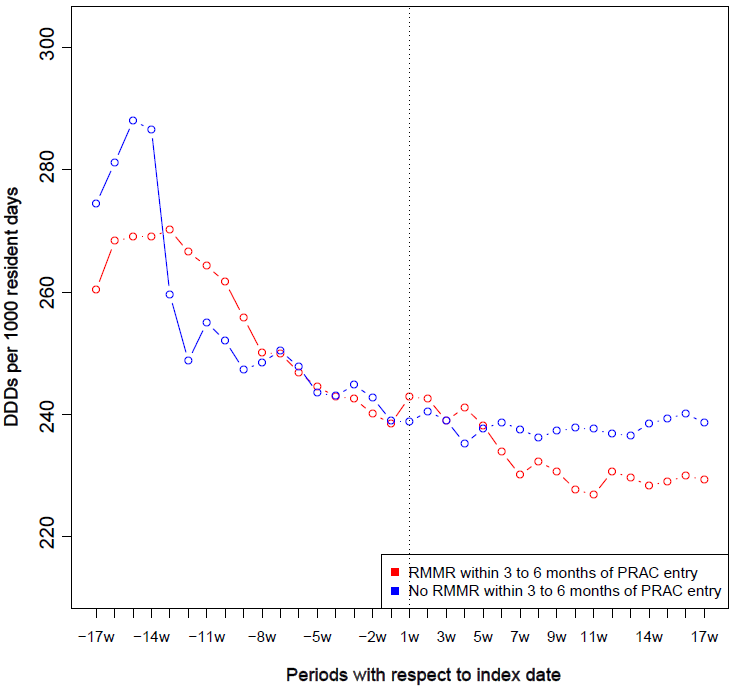


1. Benzodiazepines/zopiclone


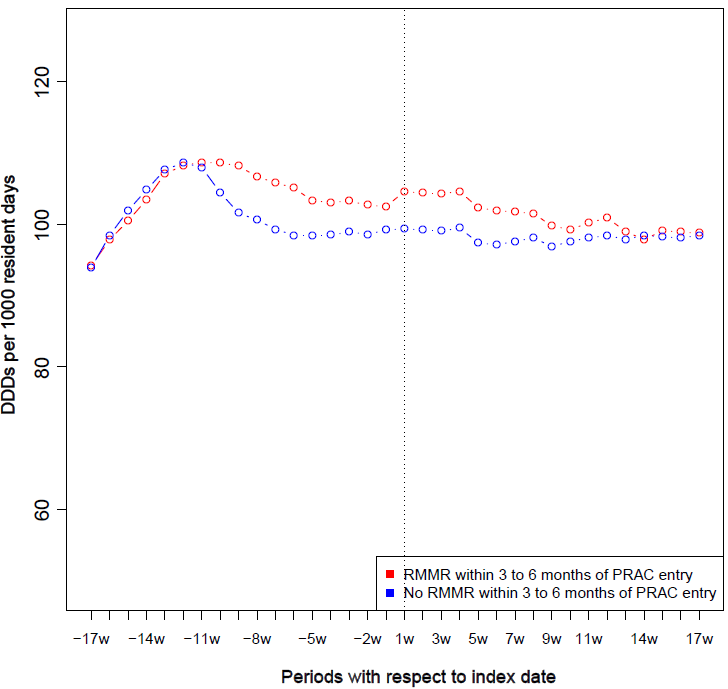


1. Antidepressants


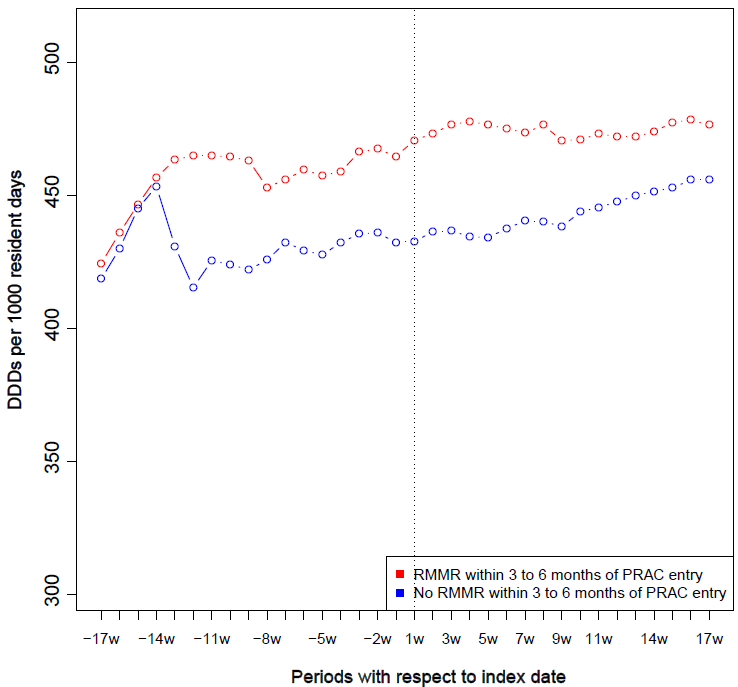


**Supplementary Figure 6**. Weekly number of DDDs per 1000 resident-days in the four months before and after the index date for individuals with an index date within 6-12 months of RACF entry

1. Benzodiazepines or zopiclone

Pre-intervention period

Wash out period

Follow-up period


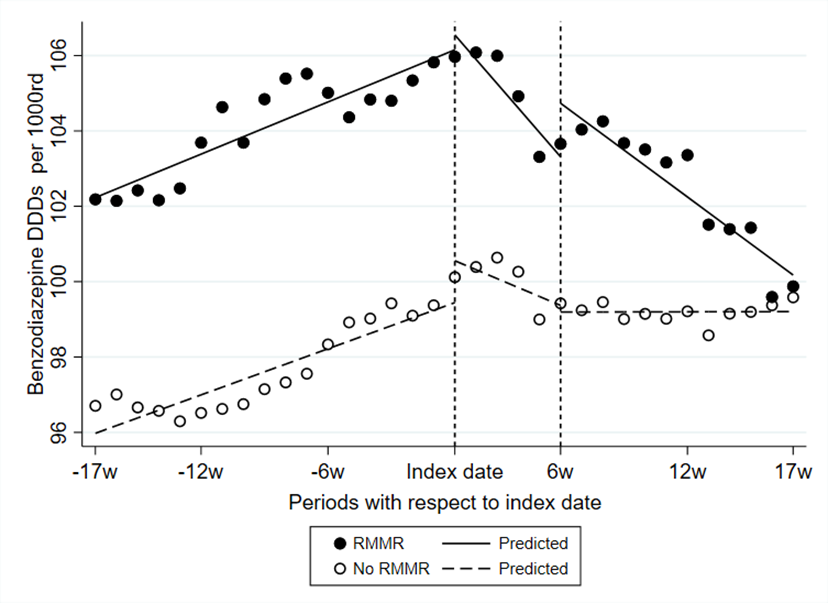


1. Calcium channel blockers (CCBs)


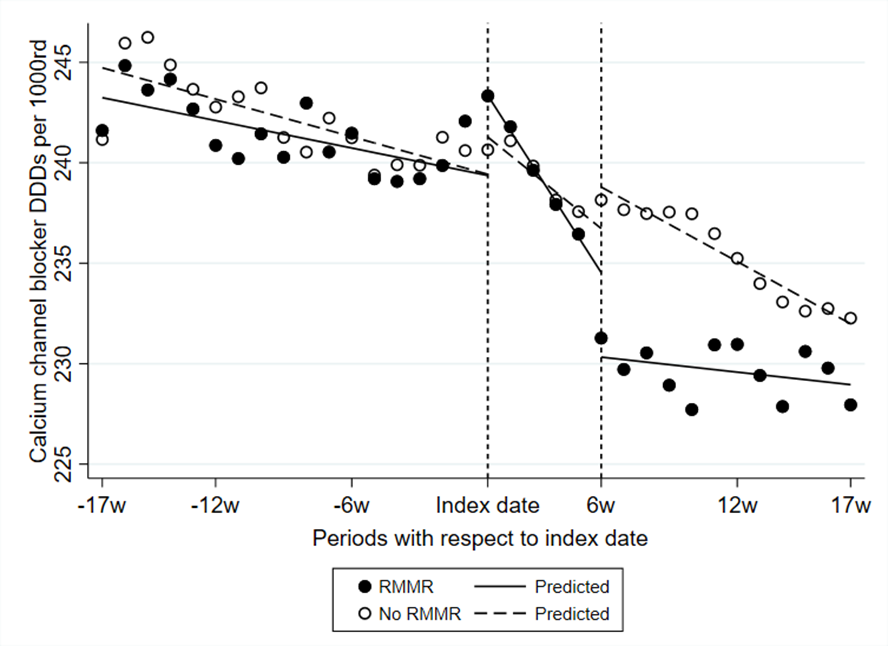


Pre-intervention period

Wash out period

Follow-up period

1. Proton pump inhibitors (PPIs)

Wash out period

Pre-intervention period

Follow-up period


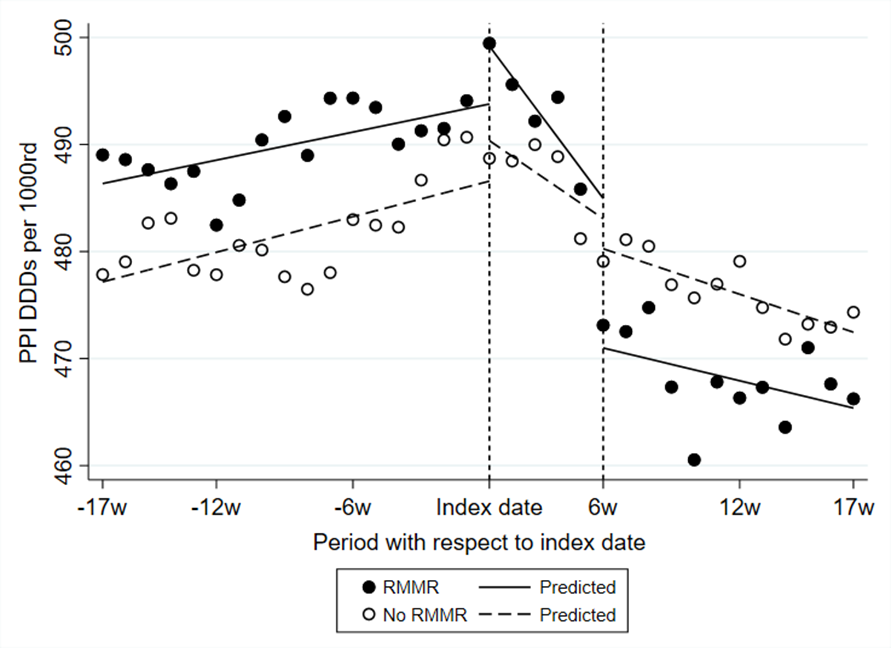


1. Antidepressants

Wash out period

Pre-intervention period

Follow-up period


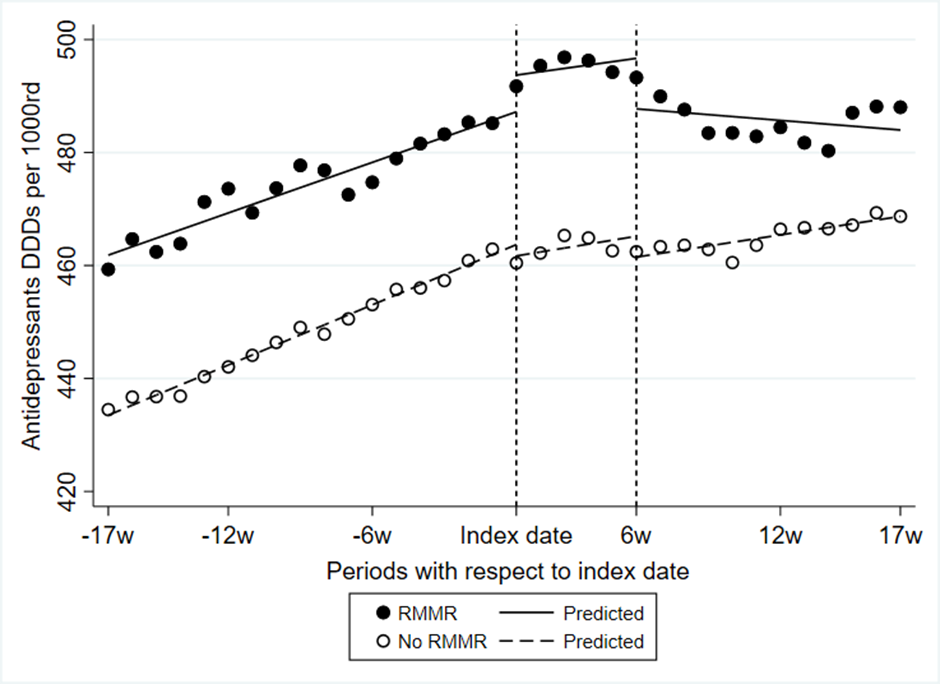


1. Antipsychotics

Follow-up period

Pre-intervention period

Wash out period


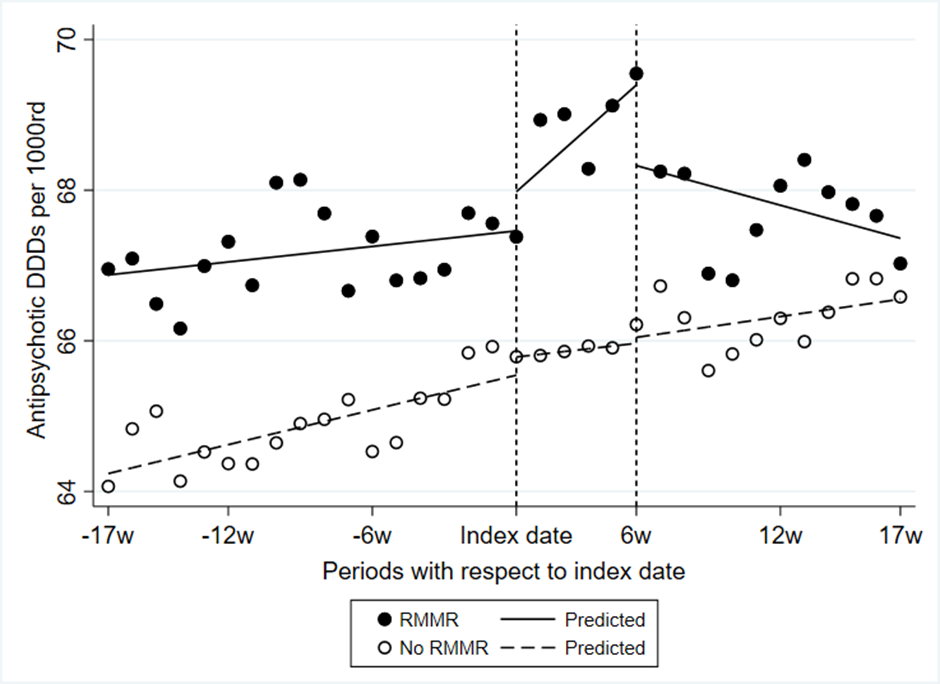


**Supplementary Figure 7**. Weekly prevalence of medicines use in the four months before and after the index date for individuals with an index date in the 6-12 months after RACF entry

1. Statins


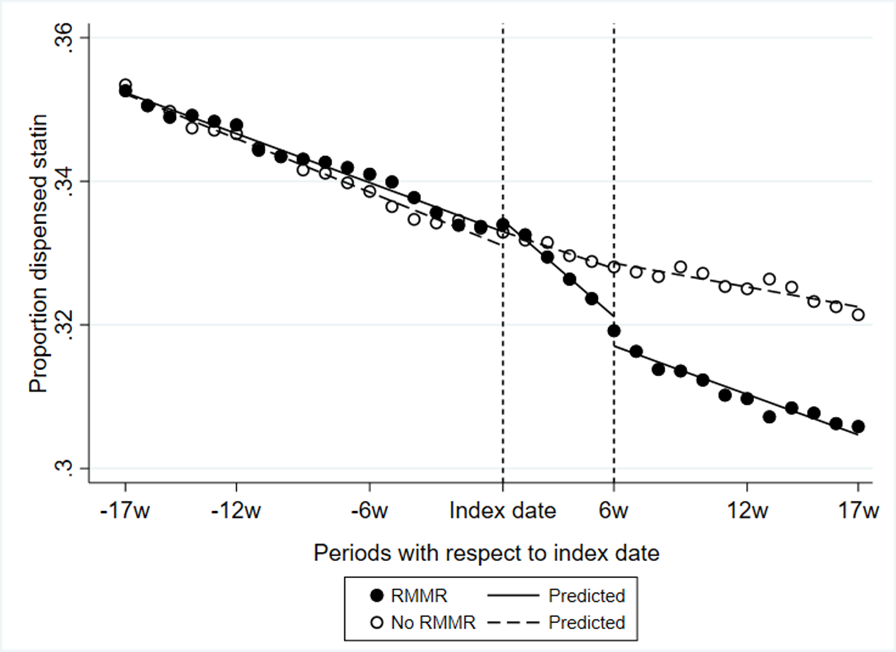


Follow-up period

Wash out period

Pre-intervention period

1. Benzodiazepines or zopiclone


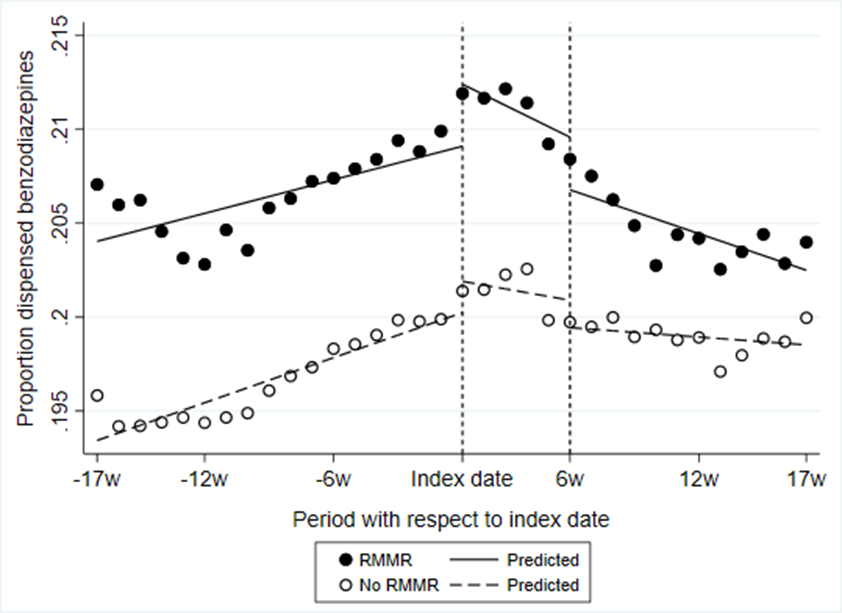


Follow-up period

Pre-intervention period

Wash out period

1. Calcium channel blockers (CCBs)


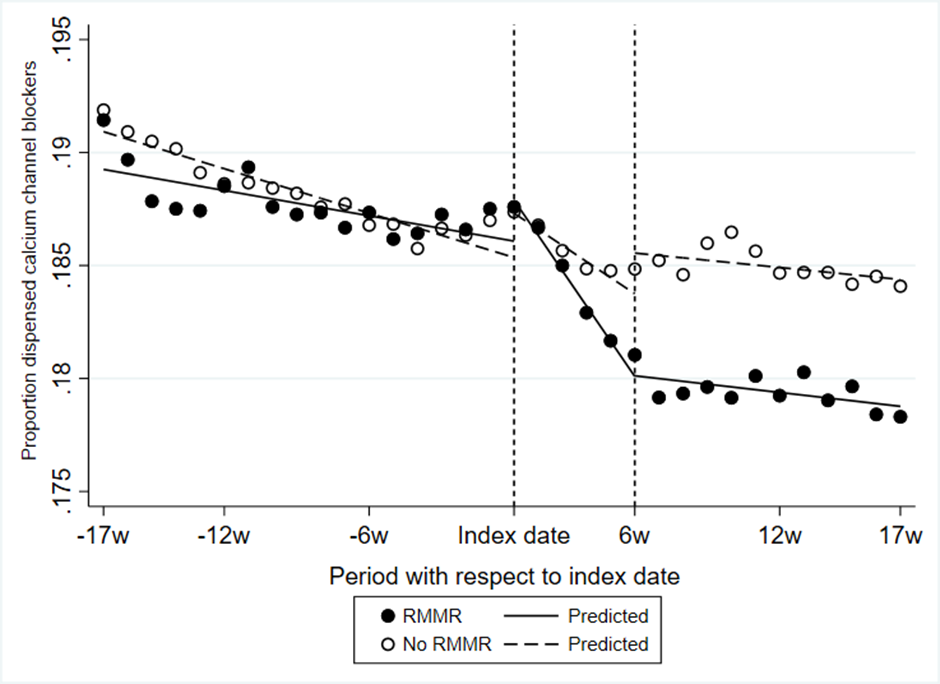


Follow-up period

Pre-intervention period

Wash out period

1. Proton pump inhibitors (PPIs)

Wash out period

Pre-intervention period

Follow-up period


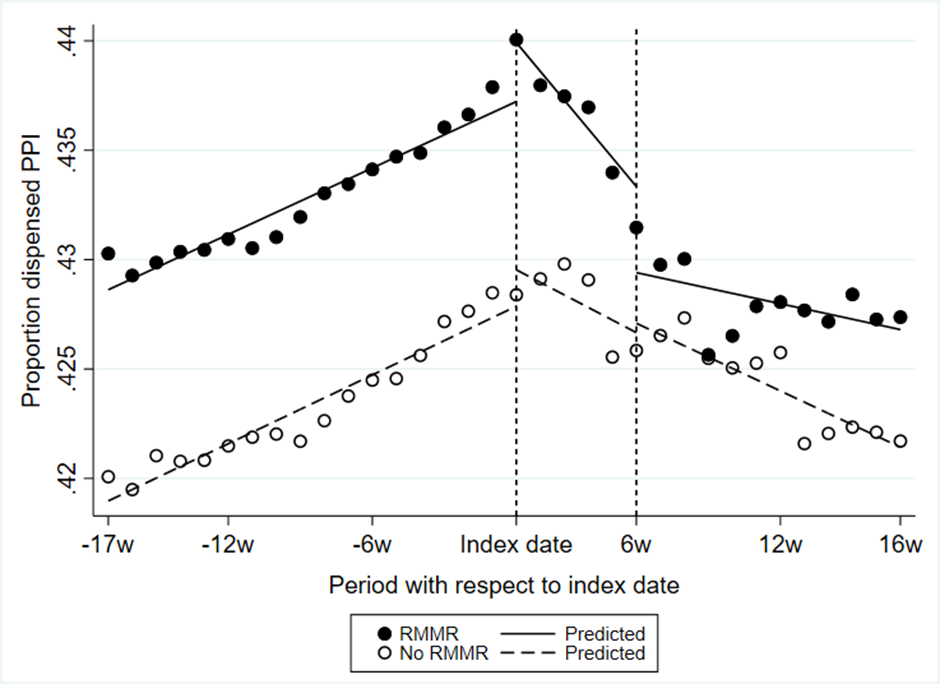


1. Antipsychotics


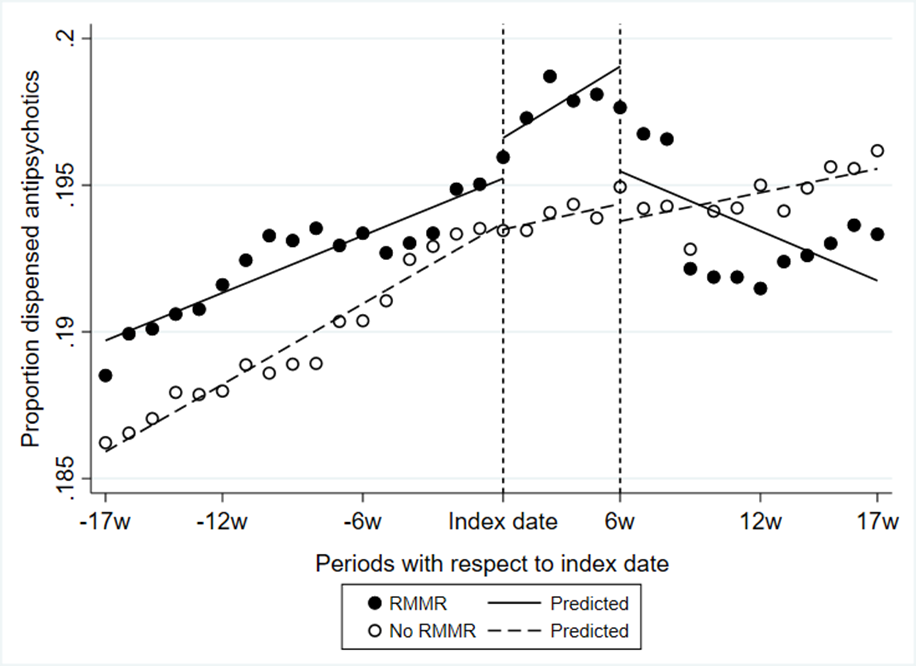


Follow-up period

Pre-intervention period

Wash out period

1. Oral anticoagulants

Follow-up period

Pre-intervention period

Wash out period


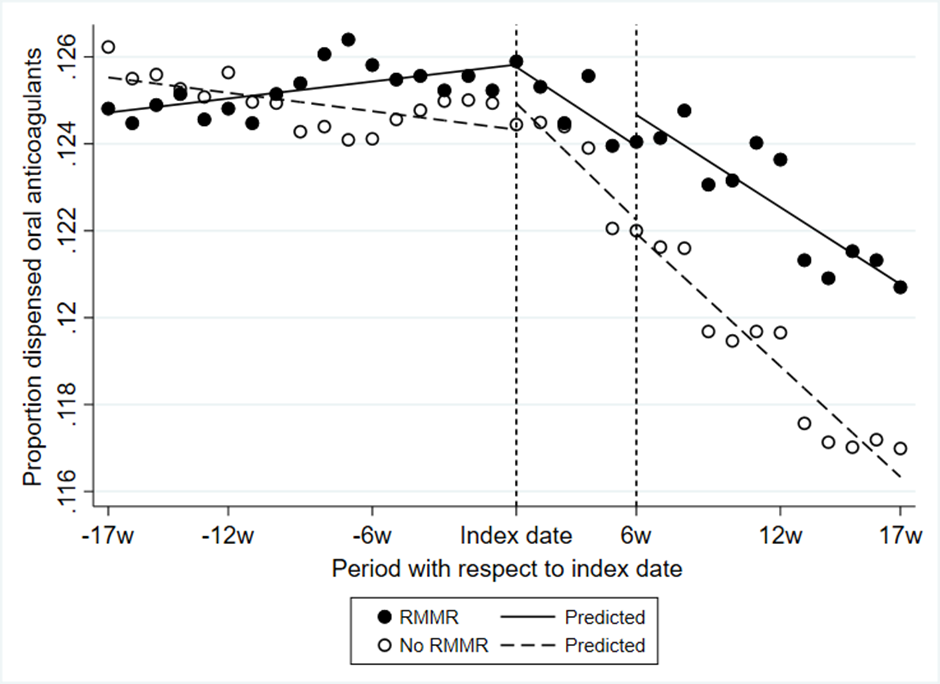

Supplement: Supplementary file 1 — Additional file 1: Supplementary Table 1. Codes used to identify medicine classes of interest, and corresponding prescription durations. Supplementary Table 2. Weekly number of defined daily doses of medicines per 1000 resident-days before and after the index date among individuals who did and did not receive an RMMR in the 0-3 months and 3-6 months after RACF entry. Supplementary Table 3. Weekly prevalence of medicines use during the study period among individuals with an index date in the 6-12 months after RACF entry. Supplementary Figure 1. Group assignment based on the date of an individual’s first RMMR relative to first entry into permanent residential aged care. Supplementary Figure 2. Time frame for assessing medicines use pre- and post-index date. Supplementary Figure 3. Time periods analyzed in the segmented regression models. Supplementary Figure 4. Weekly number of DDDs available for use per 1000 resident-days for individuals with an index date in the 0-3 months after RACF entry for medicines with possible changes in use post-RMMR compared to individuals without an RMMR. Supplementary Figure 5. Weekly number of DDDs available for use per 1000 resident-days for individuals with an index date in the 3-6 months after RACF entry for medicines with possible changes in use post-RMMR compared to individuals without an RMMR. Supplementary Figure 6. Weekly number of DDDs per 1000 resident-days in the four months before and after the index date for individuals with an index date within 6-12 months of RACF entry. Supplementary Figure 7. Weekly prevalence of medicines use in the four months before and after the index date for individuals with an index date in the 6-12 months after RACF entry. [file 12877_2022_3187_MOESM1_ESM.docx]
